# Re-emergence of human leishmaniasis in northern Italy, 2004 to 2022: a retrospective analysis

**DOI:** 10.2807/1560-7917.ES.2024.29.4.2300190

**Published:** 2024-01-25

**Authors:** Renato Todeschini, Muriel Assunta Musti, Paolo Pandolfi, Mattea Troncatti, Morena Baldini, Davide Resi, Silvano Natalini, Federica Bergamini, Giorgio Galletti, Annalisa Santi, Arianna Rossi, Gianluca Rugna, Bianca Granozzi, Luciano Attard, Valeria Gaspari, Giovanna Liguori, Margherita Ortalli, Stefania Varani

**Affiliations:** 1 Department of Public Health, AUSL Bologna, Bologna, Italy; 2 Istituto Zooprofilattico Sperimentale della Lombardia e dell’Emilia Romagna, Brescia, Italy; 3 Infectious Diseases Unit, IRCCS Azienda Ospedaliero-Universitaria di Bologna, Bologna, Italy; 4 Unit of Dermatology, IRCCS Azienda Ospedaliero-Universitaria di Bologna, Bologna, Italy; 5 Department of Medical and Surgical Sciences, Alma Mater Studiorum Università di Bologna, Bologna, Italy; 6 Unit of Microbiology, IRCCS Azienda Ospedaliero-Universitaria di Bologna, Bologna, Italy

**Keywords:** *Leishmania infantum*, visceral leishmaniasis, tegumentary leishmaniasis, environmental factors

## Abstract

**Background:**

Human leishmaniasis is a protozoan disease transmitted by sand flies and endemic in the Mediterranean region. In Italy, leishmaniasis is present in the south and the western coastal regions, with an epidemic peak detected in northern Italy in the early 1970s.

**Aim:**

To examine temporal trends, and demographic, clinical, geographical and environmental features of human leishmaniasis cases recorded by the local health unit (LHU) of Bologna, northern Italy.

**Methods:**

In this retrospective observational study, we analysed human leishmaniasis cases recorded from 2004 to 2022 within the Bologna LHU. We also conducted serological investigations for canine leishmaniasis in owned dogs living near the place of infection of human cases.

**Results:**

In total, 173 cases of human leishmaniasis were detected, and 154 cases were considered autochthonous. An increase of human cases was observed since 2004, with incidence peaks above 2 cases/100,000 inhabitants in 2013, 2018 and 2022; epidemic peaks were preceded by dry summers. Most cases lived in the plain and hilly areas less than 400 m above sea level and many resided in isolated housing, in city outskirts, and/or near uncultivated areas, watercourses and railway sections. The incidence of canine leishmaniasis did not increase in the study period.

**Conclusion:**

An epidemic of human leishmaniasis with fluctuating annual numbers of cases, probably related to environmental and climatic factors, was identified in the Bologna LHU. Understanding the risk factors and the environmental characteristics related to places of infection is crucial to evaluate the public health implications of leishmaniasis.

**Key public health message**

**What did you want to address in this study and why?**

Leishmaniasis is a neglected tropical disease caused by parasites and transmitted to humans by biting sand flies. In this study, we examined the number of people who developed human leishmaniasis in the Bologna area in northern Italy over the period 2004–22. We also examined the geographical and environmental characteristics that could be possible risk factors for human leishmaniasis, including the role of dogs as a possible reservoir of disease.

**What have we learnt from this study?**

We identified an epidemic of human leishmaniasis with fluctuating annual numbers of cases in the Bologna area, with an increase of human leishmaniasis cases since 2004, and incidence peaks in 2013, 2018 and 2022. Many cases resided in isolated housing, in city outskirts, and/or near watercourses or near railway sections. The epidemic peaks were preceded by dry summers. The incidence of canine leishmaniasis in owned dogs did not increase in the study period.

**What are the implications of your findings for public health?**

Climate change including drier summers as well as a northward spread of sand flies could have contributed to the upsurge of human leishmaniasis cases in the study area. It is important to inform the public on the potential exposure to sand fly bites in areas where the parasite circulates, and to be educated on the use of appropriate preventive measures, such as insect repellent.

## Introduction

Leishmaniasis is a protozoan disease transmitted by phlebotomine sand flies [1]. In Europe, the disease is caused by members of the genus *Leishmania*, which are parasites infecting numerous mammal species including humans; *Leishmania infantum* is the causative agent of human leishmaniasis in the Mediterranean area, where the disease exhibits a typical pattern characterised by isolated cases or small localised clusters and large epidemics are uncommon [2]. The leishmaniases are dynamic diseases because they rapidly reflect changes in transmission conditions, which are determined by environmental, demographic and comorbidity factors [3].

*Leishmania* infections in humans, though often asymptomatic, can manifest as (i) visceral leishmaniasis (VL), with a long incubation period (2–6 months), which is a serious condition characterised by irregular attacks of fever, enlarged spleen and anaemia, and often fatal if untreated; (ii) cutaneous leishmaniasis (CL), with a shorter incubation time (from 2 weeks to 6 months), consisting of benign, but sometimes disfiguring skin lesions on exposed parts of the body; and (iii) mucosal or mucocutaneous leishmaniasis (ML), which is considered rare in Europe and mainly affects mucous membranes of the nose, mouth and throat [1,4]. The CL and ML forms can be grouped into tegumentary leishmaniasis (TL).

In Italy, human leishmaniasis is a compulsory notifiable disease since 1934 [5]; leishmaniasis cases are recorded at the local health unit (LHU) level, then notifications are gathered at the regional level and subsequently centralised at the Ministry of Health. Notification data show that, while in southern Italy and the western coasts the incidence of VL has been high, the hilly areas of the Emilia-Romagna region in northern Italy have historically been affected primarily by CL, rarely by VL, and ML cases were never reported. The first documented epidemic of VL in the Emilia-Romagna region occurred during 1971 and 1972; its epicentre was in the province of Bologna, with 60 cases of VL but no reported cases of TL [6]. The epidemic occurred in the hilly areas close to Bologna municipality, where most cases carried out agricultural activity and lived in isolated houses or in small groups of buildings. After the epidemic of 1971–72, the incidence of VL in Bologna province decreased rapidly and the determinants of the outbreak have remained unexplained [7]. From 1990 to 2003, only one case of autochthonous VL was reported, with no cases of TL.

Dogs are considered the primary reservoirs of infection, even though recent evidence indicates that other domestic and wild mammals may also be reservoirs, such as cats, rodents and rabbits/hares [8]. During the 1971–72 outbreak, mass serological testing showed that 1.6% of the 8,454 owned dogs in the Bologna area were positive compared with 3.7% of 655 serologically tested persons [6,9]. Since no clinical leishmaniasis was detected in dogs in the same area, a canine reservoir for the outbreak was not proven. *Phlebotomus perfiliewi* was the main vector, with a ratio of 11:1 to *Ph. perniciosus*, showing the same predominance already reported in 1962 by Corradetti [10]. Since the early 1990s, an increasing number of autochthonous cases of canine leishmaniasis (CanL) have been detected in northern Italy, prompting the implementation of a CanL surveillance programme in public kennels of the Emilia-Romagna region since 2007 and also in owned dogs from 2011 [11].

From November 2012 to May 2013, an upsurge of VL was observed in the Bologna province; during these 6 months, 14 cases were notified [12]. After this period, the incidence of reported VL and CL increased in the LHU of Bologna as well as in other areas of the Emilia-Romagna region [13,14]. Cases of ML also emerged in the selected area [15]. This prompted us to retrospectively analyse the temporal trends of human leishmaniasis cases that were recorded by the LHU of Bologna between 2004 and 2022. We also aimed to identify environmental characteristics that are possible risk factors for this parasitic infection in the study area, including data obtained from the CanL surveillance programme of the Emilia-Romagna region.

## Methods

### Study setting

This retrospective observational study analysed cases of human leishmaniasis occurring between 1 January 2004 and 31 December 2022. The study was conducted within the LHU of Bologna, which is located in the Emilia-Romagna region, northern Italy and covers 2,915 km^2^. The area has around 890,000 inhabitants, with a population density of 304 inhabitants/km^2^. The territory is formed by Apennine mountains (790 km^2^; 27%), hills (994 km^2^; 34%) and an alluvial plain (1,131 km^2^; 39%). The climate of the area varies according to the altitude. In general, it is of the temperate sub-continental type, with hot and humid summers, cold winters, damp autumns and mild springs. Precipitation is not abundant on the plain (on average 650–800 mm/year), but increases in the hills and in the mountains, where it reaches 1,500–2,000 mm/year.

### Case definition, diagnosis and species typing

The case definition for VL in Italy, as established by the Italian Ministry of Health [16], is based on the World Health Organization (WHO) case definition [1] and includes positive serology and/or parasitology (microscopy, culture or PCR) for cases with suspected clinical signs (fever, hepatosplenomegaly, weight loss, lymphadenopathy, anaemia, leukopenia, thrombocytopenia).

Diagnosis of VL is performed on bone marrow aspirate and/or peripheral blood samples. Bone marrow smears were stained with Giemsa for microscopy examination, while serology was carried out by various methods [12,17]. A case of TL is a person showing clinical signs (skin or mucosal lesions) with parasitological confirmation (including positive histology or PCR) of the diagnosis. Diagnosis of TL is carried out by histological examination on skin or mucosal biopsies. From July 2013, real-time PCR was introduced in the diagnostic workflow of both VL (on bone marrow aspirate and/or peripheral blood) and TL (on biopsies) [14,17]. 

Species identification was performed by sequencing a region of the internal transcribed spacer-1 (ITS-1), as described previously [18].

### Human data collection

Data for this study were collected from the registry of notifiable diseases of the Epidemiology Service, Department of Public Health, Bologna, Italy. Physicians are required to report all cases of human leishmaniasis to the Public Health Service of the LHU. The LHU carries out the epidemiological investigation and collects data related to the infectious event. The registry contains clinical, diagnostic and epidemiological data. The variables of interest included: reporting year, age, sex, occupation, suspected location of infection, type of leishmaniasis, autochthonous or imported case, history of immunosuppression and travel history. 

All leishmaniasis cases who resided in the Bologna LHU and met the WHO case definition for leishmaniasis were included in the study. Cases of leishmaniasis were classified as follows: (i) autochthonous, when the suspected place of infection was within the territory of the Bologna LHU; (ii) non-autochthonous, when the suspected place of infection was outside the territory of the Bologna LHU.

### Environmental data

The epidemiological investigations conducted on the case’s home address or other place of infection for each autochthonous case concerned: (i) the altitude, (ii) the location of the housing (within an urban centre or on its outskirts or in an isolated environment; the housing was considered ‘isolated’ when part of a housing complex not exceeding 10 buildings), (iii) the presence of the following within 300 m of the place of infection, i.e. uncultivated areas (sandy or stony areas, or clay gullies), water collections (lakes, ponds or other), waterways, industrial settlements or railway tracks. The spatial range of activity of sand flies is considered to be 300 m.

### Precipitation data

Data on summer rainfall were extrapolated by Dext3r (https://simc.arpae.it/dext3r), the web app for extracting meteorological data recorded by the weather stations managed by the IdroMeteoClima Service of the Regional Agency for Prevention, the Environment and Energy (ARPAE) (https://www.arpae.it/it). 

### Canine leishmaniasis surveillance data

To consider the potential role of dogs as reservoir of the disease, data obtained from the CanL surveillance programme [11] between 2011 and 2022 were evaluated. Serological investigations were conducted in owned dogs living within 300 m from the suspected place of infection; sera from dogs were tested with an indirect immunofluorescence test [19].

### Statistical analysis

Descriptive analysis was performed by years of diagnosis, type of leishmaniasis, sociodemographic and environmental characteristics. Continuous variables are shown as mean ± standard deviation (SD) and median and interquartile range (IQR), whereas categorical variables are presented as absolute and relative frequency. Pearson’s chi-squared, Fisher’s exact, Student’s t- and Z tests were used to compare variables among type of leishmaniasis. Incidence crude rates and relative 95% confidence intervals were calculated by year, age group and sex. To identify changes in the incidence rate of human leishmaniasis, joinpoint regression and annual percentage change (APC) in rates were estimated by using the Joinpoint Regression Programme, version 4.9.1.0 (Statistical Research and Applications Branch, National Cancer Institute, United States). Relative frequency of CanL-positive dogs living within 300 m from a human case were calculated by year. P values < 0.05 were considered significant. All the analyses were performed using STATA software version 16 (StataCorp). The thematic maps with spatial distribution of the cases were provided with QGis 3.14.15-Pi (http://www.qgis.org).

## Results

From January 2004 to December 2022, 173 cases of human leishmaniasis occurred in the LHU of Bologna, northern Italy. Based on the place of infection, 154 (89%) cases were considered autochthonous, 18 (10.4%) cases were classified as non-autochthonous, and 1 (0.6%) as case of unidentified origin. Among the 18 non-autochthonous cases, the infection likely occurred outside Italy for five cases, i.e. in northern Africa (n  =  2), Albania (n  =  2), Iraq (n  =  1), or in different parts of Italy (n  =  13), including northern Italy (n  =  1), central Italy (n  =  1), southern Italy (n  =  6), Sicily (n  =  4) and territories close to the LHU of Bologna (n  =  1). 

Of the 154 autochthonous cases, 89 were VL, while 65 were TL, including four ML cases and 61 CL cases (Table 1). The four ML cases were not epidemiologically related to one other. Three of four cases (n  =  2 in 2021, n  =  1 in 2022) presented in immunocompetent individuals with granulomatous plaques or nodules in the nose and/or in the oral cavity. The fourth patient, diagnosed in 2017, was HIV-positive and presented with extensive bleeding masses in the perianal and anal mucosa. Samples that were obtained from 31 (20.1%) of the 154 autochthonous cases, including 17 CL, 3 ML and 11 VL, underwent species identification by ITS-1 typing. All samples were identified as *L. infantum*.

**Table 1:** Sociodemographic and clinical characteristics of the autochthonous cases of human leishmaniasis recorded by the local health unit of Bologna, northern Italy, January 2004–December 2022 (n = 154)

Characteristics	Visceral leishmaniasis		Tegumentary leishmaniasis		Total		p value
	n	%	n	%	n	%	
Total	89	57.79	65	42.21	154	100	
Sex							
Male	74	83.15	47	72.31	121	78.57	0.105
Female	15	16.85	18	27.69	33	21.43	
Age (years)							
Mean (SD)	53.60 (23.09)		55.49 (20.08)		54.40 (21.82)		0.596
Median (IQR)	59 (45–70)		57 (45–70)		59 (45–70)		0.668
0–2	9	10.11	2	3.08	11	7.14	0.420
3–9	0	0	2	3.08	2	1.30	
10–19	1	1.12	0	0	1	0.65	
20–29	3	3.37	1	1.54	4	2.60	
30–39	7	7.87	6	9.23	13	8.44	
40–49	12	13.48	9	13.85	21	13.64	
50–59	13	14.61	16	24.62	29	18.83	
60–69	21	23.60	12	18.46	33	21.43	
70–79	15	16.85	10	15.38	25	16.23	
80–89	7	7.87	7	10.77	14	9.09	
≥ 90	1	1.12	0	0	1	0.65	
Comorbidities							
0	66	74.16	57	87.69	123	79.87	0.039*
≥ 1	23	25.84	8	12.31	31	20.13	0.039*
Chronic viral hepatitis	0	0	1	1.54	1	0.65	0.240
Other chronic liver disease	1	1.12	0	0	1	0.65	0.376
Renal failure with transplantation	1	1.12	0	0	1	0.65	0.376
Sjogren's disease	1	1.12	0	0	1	0.65	0.376
Asbestosis	0	0	1	1.54	1	0.65	0.240
Sarcoidosis	1	1.12	0	0	1	0.65	0.376
COPD	0	0	1	1.54	1	0.65	0.240
Hypertensive heart disease	0	0	1	1.54	1	0.65	0.240
HIV-positive	2	2.25	1	1.54	3	1.95	0.753
Iatrogenic immunosuppression	3	3.37	0	0	3	1.95	0.135
Rheumatoid arthritis	2	2.25	2	3.08	4	2.60	0.749
Diabetes	4	4.49	2	3.08	6	3.90	0.655
Alcohol use disorder	7	7.87	0	0	7	4.55	0.021*
Cancer	11	12.36	0	0	11	7.14	0.003*
Death							
Case fatality rate	10	11.24	1	1.54	11	7.14	0.021*
Occupational activities							
Working environment mainly outdoors	16	17.98	9	13.85	25	16.23	0.493
Working environment mainly indoors	24	26.97	26	40.00	50	32.47	0.088

COPD: chronic obstructive pulmonary disease; IQR: interquartile range; SD: standard deviation.

P values < 0.05 are considered significant and are indicated with an asterisk.

Pearson’s chi-squared and Fisher’s exact tests were used to compared sex and age classes among type of leishmaniasis, respectively. Student’s t-test was used to compared mean and median age and a Z-test was used to compared occupational activities, comorbidities and case fatality rate.

### Incidence of human leishmaniasis

**Figure 1:**
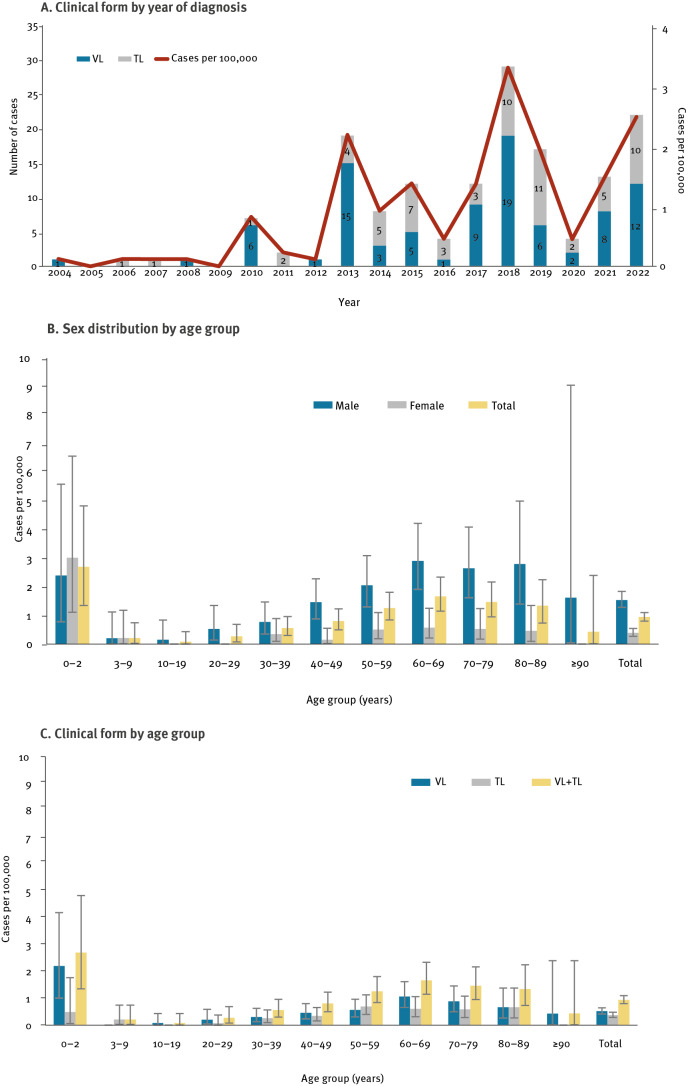
Incidence rate of autochthonous cases of human leishmaniasis by clinical form and year of diagnosis (A), age and sex (B) and age and clinical form (C), local health unit of Bologna, northern Italy, January 2004–December 2022 (n = 154)

TL: tegumentary leishmaniasis; VL: visceral leishmaniasis.

In the period 2004–22, a significant increase in autochthonous cases of human leishmaniasis was observed (APC: 18.3, p value < 0.001). The overall incidence rate of autochthonous cases was 0.9 cases per 100,000 inhabitants per year; the highest rates were observed in 2018 and in 2022 (3.3 and 2.5 cases per 100,000, respectively, Figure 1A). The overall incidence rate was significantly higher in males than females (1.5 vs 0.4 per 100,000, respectively, Figure 1B). In addition, the incidence rate was significantly higher in the age group 0–2 years compared with age groups ranging from 3 to 49 years, with an average incidence of 2.7 and 0.4 cases per 100,000 per year, respectively (Figure 1C); in the age group 0–2 years, however, both sexes were equally affected (Figure 1B). No age differences were detected between VL and TL (Figure 1C). The mean age of cases was 54 years (SD: 23.1) for VL and 55 years (SD: 20.1) for TL (p value: 0.596). No differences were found in the mean ages when excluding immunocompromised individuals (51 ± 24.5 and 54 ± 20.1 years, for VL and TL, respectively, p value: 0.474). 

A. Numbers on the histogram represent cases of human leishmaniasis for respective clinical forms of leishmaniasis.

B and C. Bars represent 95% confidence intervals.

### Comorbidities, case fatality and occupation 

Leishmaniasis was linked to chronic disease or immunosuppression in 31 cases (20.1%, Table 1). Among the 89 cases of VL, 11 cases (12.4%) were affected by cancer, seven cases (7.9%) by alcohol use disorder and three cases (3.4%) by iatrogenic immunosuppression; cancer and problematic alcohol use were more frequent in the VL group compared with the TL group (p value: 0.003 and 0.021, respectively). Moreover, two of 89 VL cases (2.2%) as well as one of 65 TL cases (1.5%) were HIV-positive. Eighty-one percent of leishmaniasis cases with at least one of the above-mentioned chronic conditions were aged 50 years or over. Ten VL cases died; the case fatality rate for VL was 11.2%. 

The occupational activities of the cases were divided into two groups: activities carried out mainly indoors vs mainly outdoors. There was a lower risk (not significant) of VL (27%) vs TL (40%) for cases who carried out mainly indoors activities.

### Geographic distribution and environmental characteristics

**Figure 2:**
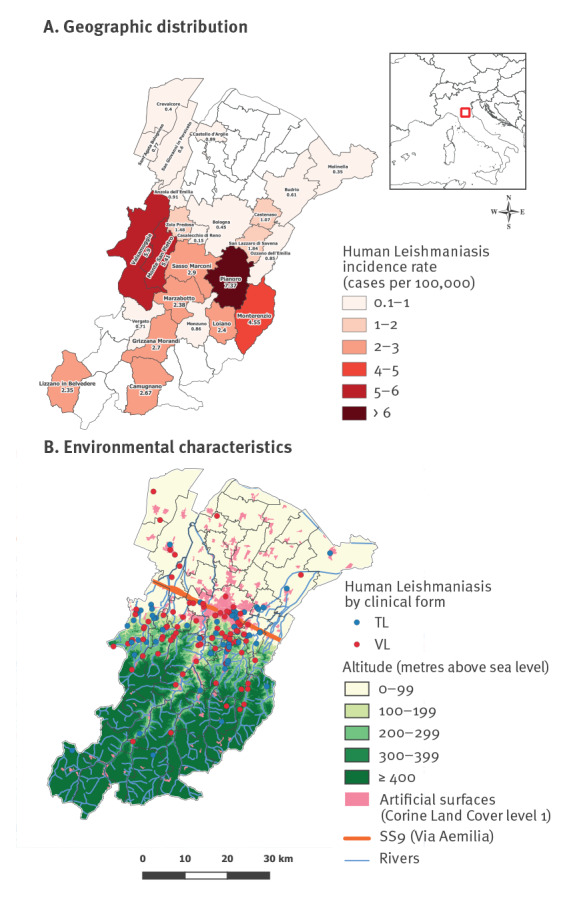
Geographic distribution of autochthonous cases of human leishmaniasis based on different municipalities (A) and environmental characteristics (B), local health unit of Bologna, northern Italy, January 2004–December 2022 (n = 154)

TL: tegumentary leishmaniasis; VL: visceral leishmaniasis.

**Figure 3:**
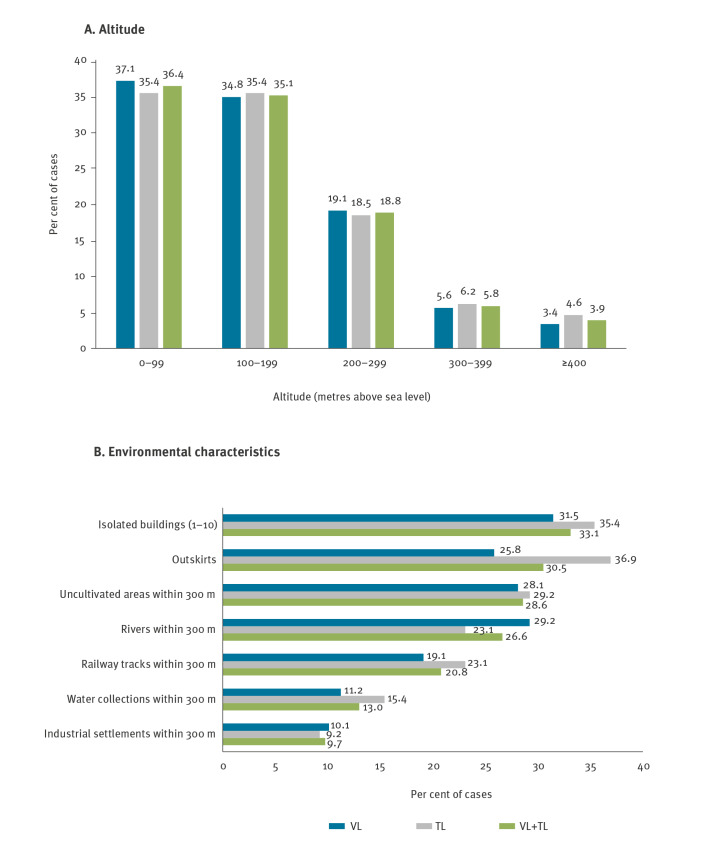
Distribution of autochthonous cases of human leishmaniasis based on altitude above sea level (A) and on environmental characteristics (B), local health unit of Bologna, northern Italy, January 2004–December 2022 (n = 154)

TL: tegumentary leishmaniasis; VL: visceral leishmaniasis.

Different municipalities in the Bologna territory exhibited different incidence rate of human leishmaniasis (Figure 2A). Cases of leishmaniasis were distributed mostly in areas below 400 m above sea level (masl) (n  =  148, 96.1%), six cases (3.9%) resided between 400 and 800 masl, no cases were detected over 800 masl (Figure 2B, Figure 3A). Environmental characteristics of the place of infection are shown in Figure 3B; the most common features were the presence of uncultivated areas (n  =  44, 28.6%), residing in isolated buildings (n  =  51, 33.1%) or in the outskirts of an urban area (n  =  47, 30.5%), residing near watercourses (n  =  41, 26.6%) or near railway tracks (n  =  32, 20.8%).

Corine Land Cover is a detailed and harmonised dataset about land cover and land use of the European continent, organised in 44 categories. It is a product of the European Union's Copernicus Land Monitoring Service information (https://land.copernicus.eu/en; https://groupware.sinanet.isprambiente.it/uso-copertura-e-consumo-di-suolo/library/copertura-del-suolo/corine-land-cover; data accessed on 21 Dec 2023).

Numbers with each bar represent the percentage for each clinical form. For each case of leishmaniasis, one or more answers are possible.

**Figure 4:**
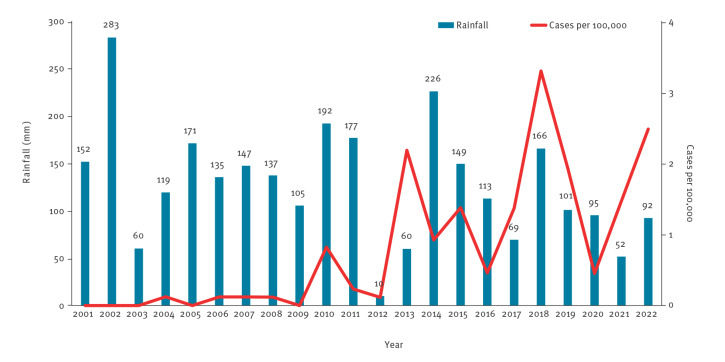
Total rainfall and curve of leishmaniasis cases during the summer months, local health unit of Bologna, northern Italy, June, July and August, 2001–2022

Data on summer rainfall collected in the LHU of Bologna from 2001 to 2022 were analysed; dry summers (2003, 2012, 2017 and 2021) preceded the increase in annual cases (2004) and the peaks (2013, 2018 and 2022) of the present outbreak (Figure 4).

Data on summer rainfall were extracted by Dext3r (https://simc.arpae.it/dext3r). The weather stations for reference are located in the city of Bologna, 80 m above sea level. Numbers on the tops of each histogram represent mm of rainfall by year during June, July and August.

### Canine leishmaniasis

From 2011 to 2022, 1,422 owned dogs living within 300 m from the suspected place of infection of 82 cases of human leishmaniasis were sampled within the LHU of Bologna; the positive rate for CanL varied between 0% and 3.3%, with no significant difference between years (Table 2).

**Table 2:** Analysis of canine leishmaniasis in owned dogs living within 300 m from 82 human cases, local health unit of Bologna, northern Italy, 2011–2022 (n  =  1,422)

Year of diagnosis	Human cases considered	Dogs sampled^a^			Per cent positive	95% CI
		Total	Negative^b^	Positive^c^		
2011	0	0	0	0	0	0–100
2012	1	28	28	0	0	0–12.34
2013	13	489	481	8	1.64	0.71–3.20
2014	8	133	131	2	1.50	0.18–5.33
2015	5	112	111	1	0.89	0.02–4.87
2016	4	114	113	1	0.88	0.02–4.79
2017	6	61	59	2	3.28	0.40–11.35
2018	17	135	132	3	2.22	0.46–6.36
2019	13	255	249	6	2.35	0.87–5.05
2020	1	7	7	0	0	0–40.96
2021	7	41	40	1	2.44	0.06–12.86
2022	7	47	47	0	0	0–7.55
Total	82	1,422	1,398	24	1.69	1.08–2.50

CI: confidence interval.

^a^ Owned dogs living within 300 m from the identified human cases.

^b^ Dogs that tested negative with an indirect immunofluorescence test (IF) (titre <  1:160).

^c^ Dogs that tested positive with an IF (titre ≥  1:160).

## Discussion

This study provides a 19-year analysis of the epidemiological patterns, including case characteristics, presence of infected dogs and environmental features of human leishmaniasis in the LHU of Bologna in northern Italy. Since 2004, we observed a significant increase in autochthonous cases of human leishmaniasis caused by *L. infantum*, with peaks of incidence above 2 cases per 100,000 inhabitants in 2013, 2018 and 2022.

In line with published evidence [1,20], most cases with leishmaniasis were male, with no age differences between VL and TL. The incidence rate of leishmaniasis was higher in the age group 0–2 years (essentially because of VL) than in the age groups including those between 3 and 49 years, similar to the VL epidemic of 1971–72 [6]. An increased number of cases was also found in the 60–69-year age group, possibly because of a combination of more outdoor activities after retirement and the gradual deterioration of the immune defences with age.

In this epidemic with fluctuating annual numbers of cases, three cases occurred in HIV-positive individuals (1.9%), in contrast with most data from southern Europe showing high rates of *Leishmania*-HIV coinfection [1]. However, an outbreak of human leishmaniasis that occurred in Madrid (Spain) in 2009–12 also reported a low HIV/*Leishmania* co-infection rate [21]. Furthermore, leishmaniasis cases in HIV-infected individuals declined throughout Italy since 2001 because of the introduction of antiretroviral therapies [2]. Our findings show that VL cases were associated with other potentially immunosuppressive diseases, such as cancer (12.4%), iatrogenic immunosuppression (4.5%) and alcohol use disorder (7.9%). The improvement in healthcare, diagnostic methods and anti-leishmanial treatment has most likely played a role in the reduction of VL lethality compared with that of the 1971–72 epidemic [6].

Almost all autochthonous cases of leishmaniasis (96.1%) were distributed in the plain and hilly areas below 400 masl, with no difference between VL and TL cases. Seventy percent of autochthonous cases lived south of the main road crossing the Emilia-Romagna region, the so-called Via Aemilia (SS9), 20.8% lived in the town of Bologna and 9.1% lived in the plain area north of the SS9; this is in contrast with the 1971–72 epidemic, when as many as 96.7% of cases lived south of the SS9, none lived in Bologna town and only 3.3% lived in the plain area north of the SS9 [6]. This distribution, with no difference between VL and TL cases, suggests that human leishmaniasis has extended northward. In line with this observation, recent studies in the selected area displayed an increased density of sand flies and their ability to colonise the northern plain of Italy, which was previously considered unsuitable for these insects [22,23]. As a hypothesis to explain the northward spread of human leishmaniasis, animal reservoirs could have followed the waterways as well as the railway sections northward, encountering a higher number of sand flies in the plain than in the past because of climate change. In support of this hypothesis, we observed that 26.6% of human leishmaniasis cases were identified near the waterways and 20.8% near the railway sections, suggesting that, in an area as densely populated as that of the LHU of Bologna, these could be ecological niches and dispersal corridors for wild mammals and sand flies [24–26].

In the current epidemic with fluctuating annual numbers of cases, many cases resided in isolated homes (33.1%) and/or on the outskirts of a built-up area (30.5%), similar to the distribution of cases during the 1971–72 epidemic [6]. This may reflect the ecological niche of both sand flies and mammals, which are commonly found in rural and semi-natural environments [22]. Furthermore, all the examined environmental data were broadly similar for VL and TL cases. This observation suggests that the reservoir(s) of infection and the vectors are largely the same for VL and TL.

Ongoing climate change including a rise in the average annual temperature [27] could contribute to the variability in the annual density pattern of sand flies, likely affecting the vector’s biological cycle [28]; the exceptional spring-summer dryness occurring in northern Italy in 2017 has been associated with an increased sandfly abundance [22]. Similarly to the 1971–72 VL epidemic in Bologna, which was preceded by the driest summer of the 20 years prior [6], dry summers preceded the epidemic onset and the major peaks of the current epidemic.

In the considered period (2011–22), CanL did not seem to increase among dogs, in contrast to the registered upsurge of human leishmaniasis cases. Nevertheless, data on owned dogs are scant and do not allow strong conclusions to be drawn on this matter. While dogs do not appear to be the primary cause of the re-emerging leishmaniasis threat, efforts to maintain low prevalence in this species could contribute to the parasite control. Although immunisation can prevent the development of severe disease, it does not protect dogs from infection. Therefore a comprehensive control strategy must be employed, including use of topical repellents [29].

Molecular studies of the *Leishmania* parasites in the selected area, including multilocus microsatellite typing as well as the amplification of *cpb E/F*-gene and *k26*-gene suggest that the parasitic strain causing VL and the strain isolated from *Ph. perfiliewi* are distinct from the strain circulating in dogs [18,30]; therefore, other mammals could play a role in local transmission. In line with this hypothesis, leishmanial DNA was recently detected in ca 6% of wild animals (including roe deer, hares, foxes and wolves) that were tested in the Emilia-Romagna region [31], as well as in 10–13% of peridomestic rodents [32]. The epidemic of human leishmaniasis with fluctuating annual numbers of cases that we observed in northern Italy shares some characteristics with the outbreak of this parasitic disease that occurred in Madrid, Spain in 2009–12 [21]. Similar to the ecological dynamics in northern Italy [22,23,30], the Spanish outbreak reported a high density of sand flies as well as a low infection rate in dogs [33]. Hares were identified as pathogen reservoirs in the Spanish epidemic [33], while in the current epidemic in Bologna, an animal reservoir has not been clearly identified, but several wild and peridomestic animals exhibit high rate of *Leishmania* infection [31,32]. 

Since there is no vaccine against human leishmaniasis and control measures against sand flies are not available, it would be essential to identify mammals’ reservoirs and environmental factors related to *Leishmania* infection. Considering the latter aspect, this study emphasises the importance of examining environmental data related to autochthonous human cases in endemic regions. The public needs to be aware of the potential exposure to sand fly bites in areas in which the parasite circulates, and to be educated in the use of appropriate preventive measures, such as mechanical and chemical insect repellents. Identifying the parasite reservoirs in the selected area would allow the control of their presence/density, for example, by intensifying rodent control in the case rats would be demonstrated as *Leishmania* reservoirs, as recently suggested [32]. A number of factors, including environmental transformation related to human activities and climate change could contribute to the increasing number of leishmaniasis cases in this area. The extensive use of molecular tools for identification and typing of the *Leishmania* parasite in humans, potential animal reservoirs and vectors will likely contribute to understand the atypical ecological cycle of the parasite in the selected area.

This study has some limitations. The data are collected from a limited geographic area, and the results cannot give information about the leishmaniasis scenario in the entire country. Furthermore, diagnostic tools to detect human leishmaniasis have improved significantly since 2014, with the introduction of molecular diagnosis after the recognition of the 2013 outbreak of VL in the study area [12,17]. The enhanced sensitivity of diagnostic methods as well as the increased awareness about this parasitic disease among clinicians may have contributed to the increase in case detection.

## Conclusions

An epidemic of human leishmaniasis with fluctuating annual numbers of cases has occurred in the Bologna LHU, northern Italy. The reasons for the increased incidence of human leishmaniasis in the selected area are not well known, but could be related to climate change including drier summers, with an increase and a northward spread of sand flies, or the presence of different and potentially multiple reservoirs of the parasite, which perhaps are also increasing and expanding. Thus, it would be important to make efforts to identify the parasite reservoirs in the selected area to control their density. This study also emphasises the importance of examining environmental data related to autochthonous human cases in endemic regions to create awareness and educate the public on preventive measures.
